# Enhanced Detection of Leishmania Parasites in Microscopic Images Using Machine Learning Models

**DOI:** 10.3390/s24248180

**Published:** 2024-12-21

**Authors:** Michael Contreras-Ramírez, Jhonathan Sora-Cardenas, Claudia Colorado-Salamanca, Clemencia Ovalle-Bracho, Daniel R. Suárez

**Affiliations:** 1 Facultad de Ingeniería, Pontificia Universidad Javeriana, Bogotá 110231, Colombia; contreras.michael@javeriana.edu.co (M.C.-R.); j_sora@javeriana.edu.co (J.S.-C.); clemencia-ovalleb@javeriana.edu.co (C.O.-B.); 2 Hospital Universitario Centro Dermatológico Federico Lleras Acosta ESE, Bogotá 110231, Colombia; jefedocencia@dermatologia.gov.co

**Keywords:** cutaneous leishmaniasis, direct smear examination, preprocessing, segmentation, machine learning, grid search

## Abstract

Cutaneous leishmaniasis is a parasitic disease that poses significant diagnostic challenges due to the variability of results and reliance on operator expertise. This study addresses the development of a system based on machine learning algorithms to detect *Leishmania* spp. parasite in direct smear microscopy images, contributing to the diagnosis of cutaneous leishmaniasis. Starting with acquiring and labeling 500 images, an experimental design was implemented, including preprocessing and segmentation techniques such as Otsu, local thresholding, and Iterative Global Minimum Search (IGMS) to improve parasite detection. The phenotypic features of the parasites were extracted, focusing on morphology, texture, and color. Machine learning models (ANN, SVM, and RF) optimized through Grid Search were applied for classification. The model with the best results was a Support Vector Machine (SVM), achieving a sensitivity of 91.87% and a specificity of 89.21% at the crop level. Compared with previous studies, these results highlight the relevance and consistency of the methodology used, supporting the initial hypothesis. This suggests that machine learning techniques offer a promising path toward improving the diagnosis of cutaneous leishmaniasis.

## 1. Introduction

Leishmaniasis is a globally widespread parasitic disease caused by infection with parasites of the genus *Leishmania*, transmitted through infected sandflies’ bite. According to data from the World Health Organization (WHO), it represents a significant public health burden, with between 700,000 to 1 million new cases and around 70,000 deaths reported annually worldwide. This disease affects regions in Asia, Africa, the Americas, and the Mediterranean [1]. It manifests in three primary clinical forms: cutaneous leishmaniasis, mucocutaneous leishmaniasis, and visceral leishmaniasis, each varying in severity. However, cutaneous leishmaniasis is the most prevalent form globally, and direct smear microscopy is the diagnostic method most used for this type of leishmaniasis. This method presents significant challenges due to its dependence on operator expertise and sensitivity variability, ranging from 40% to 90% [2]. More advanced techniques, such as Polymerase Chain Reaction (PCR) and serological tests, exist but are costly and not always available in primary care settings where a high incidence is common.

In recent years, several researchers have adopted approaches that combine artificial intelligence techniques and image processing in the field of microscopy to detect, classify, and diagnose pathologies. Although there are not many studies directly related to leishmaniasis compared to those conducted on similar tropical diseases such as malaria and chagas, these share similarities in the collection, processing, and analysis of samples.

Those similitudes allow the implementation of comparable techniques in the context of leishmaniasis. These techniques include basic algorithms and traditional methods focused on image acquisition, preprocessing using color spaces such as RGB and HSV, noise reduction filtering techniques, segmentation based on thresholding techniques, and classification using rule-based sets. There are also approaches based on feature extraction and classification using machine learning algorithms. These methods involve algorithms such as clustering, decision trees, adaboost, artificial neural networks (ANN), and support vector machines (SVM). The features used to train the models include information about color, texture, and morphology [3,4,5,6].

Studies focusing on leishmaniasis have centered on the segmentation, detection, and classification of the *Leishmania* parasite. For example, Zare [7] implemented machine learning techniques and algorithms such as Viola-Jones, achieving a sensitivity of 71% and a specificity of 52% in detecting amastigotes. Additionally, relevant research in the field of cutaneous leishmaniasis, such as Abdelmula’s study [8], evaluated five pre-trained deep-learning models and achieved an average accuracy of 99.14% when classifying between positive and negative cutaneous leishmaniasis images. Gonzalves [9] applied deep learning techniques for *Leishmania* parasite segmentation, reaching a sensitivity of 72.2%. These studies demonstrate the success of different approaches in parasite detection, offering a promising solution to improve the accuracy of pathology diagnosis in medical images.

Therefore, this research is justified by improving the efficiency and sensitivity of cutaneous leishmaniasis diagnosis, especially in primary care settings. Our hypothesis is based on evidence that machine learning algorithms (such as the ones used in [10,11,12]) can play a crucial role in detecting *Leishmania* spp. parasites in microscopy images, which could lead to a more accurate diagnosis. In this study, we developed and evaluated a machine learning algorithm that detects the main structures of the *Leishmania* spp. parasite based on segmented parasite features. The extracted features consisted of morphology, color, and texture phenotypic descriptors.

## 2. Methodology

**Figure 1:**
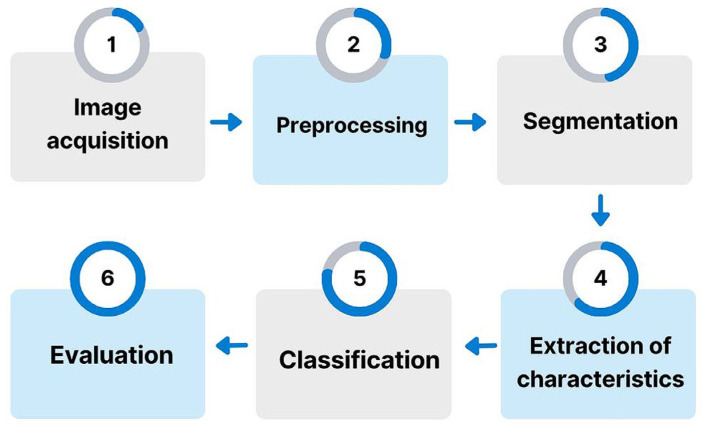
Project methodology diagram.

The general methodology has six stages (see Figure 1). The first stage involved acquiring and consolidating a database containing images of direct smear examinations for cutaneous leishmaniasis. The second stage focused on preprocessing to reduce noise in the images. The third stage involved segmentation and allowed for extracting regions of interest as potential parasite candidates. The fourth stage focused on extracting a feature set representing the phenotypic structures of the *Leishmania* spp. parasite. In the fifth stage, machine learning methods were implemented to classify the images into two categories (parasite or non-parasite). Finally, the sixth stage involved evaluation using statistical validation metrics to analyze the results obtained by the system.

### 2.1. Image Acquisition

A total of 50 fully anonymized Giemsa-stained direct smear slides were obtained from male and female patients diagnosed with cutaneous leishmaniasis, representing both acute and chronic stages of the disease. The samples were collected from diverse endemic regions across Colombia, including urban and rural areas, ensuring sufficient demographic variability. This diversity reflects the range of clinical presentations commonly encountered in real-world diagnostic settings and strengthens the representativeness of the dataset for machine learning applications. The slides were provided by the Hospital Universitario Centro Dermatológico Federico Lleras Acosta (HUCDFL) in Bogotá, Colombia.

The inclusion criterion required the presence of at least five parasites per microscopic field, a standard validated by microscopy experts. To ensure reproducibility, the digitization of the slides followed a standardized protocol developed in collaboration with specialists in clinical diagnostics. Each slide was imaged using a 100× oil immersion objective (PlanC N 100×/1.25 Oil, FN 22) mounted on an Olympus CX43 microscope (Olympus, Tokyo, Japan) equipped with an integrated Olympus EP50 camera system. The imaging process was controlled via the EPView software (V1.3), and consistent lighting conditions were maintained using a lux meter (Model 407026, Extech, Street Pittsburgh, PA, USA). Each field of view was captured at a resolution of 2592 × 1944 pixels, ensuring high-quality images suitable for subsequent processing and analysis.

To facilitate the use of the images in machine learning models, parasite annotation was performed using the LabelImg software (V1.8.6). This process involved the creation of precise bounding boxes around individual parasites, allowing for the extraction of height, width, and positional coordinates (x, y) for each parasite. The annotation process was conducted in collaboration with experts in cutaneous leishmaniasis diagnosis, ensuring the accuracy and reliability of the dataset. The resulting annotations were saved in JSON format, directly linked to their corresponding image files. This comprehensive and standardized process consolidated a high-quality dataset that reflects a wide range of clinical and demographic characteristics. The dataset is a robust foundation for the study’s preprocessing, segmentation, and classification stages.

### 2.2. Preprocessing

In the absence of a protocol specifically tailored for preprocessing cutaneous leishmaniasis images, this study adopted preprocessing techniques inspired by related research on diseases with comparable diagnostic methods, such as malaria and Chagas [10,11,12,13,14]. The preprocessing stage consisted of two main processes: color space decomposition and applying noise reduction and contrast enhancement techniques.

In the first step, the original images were decomposed into RGB and HSV color spaces and a Grayscale conversion. These color spaces were selected for their proven effectiveness in highlighting the distinctive features of microscopic images. Seven sub-images were generated for each original image, corresponding to the individual channels (R, G, B, Grayscale, H, S, V). This approach enabled the exploration of critical color variations to identify parasites and enhance the visibility of their phenotypic features.

Subsequently, three filtering techniques were applied for noise reduction. Median filtering was used to eliminate small noise points while preserving the sharpness of parasite structure edges, which is crucial for maintaining their morphological integrity. Gaussian filtering was applied to smooth the images, reducing random noise and improving the overall clarity of the image. Morphological closing, combining dilation followed by erosion, was used to close small gaps within visible structures in the image, enhancing the continuity of parasite shapes. For contrast enhancement, two techniques were tested to improve the distinction between darker regions (representing parasite structures) and lighter backgrounds. Laplacian filtering was employed to highlight edges and better define the phenotypic features of the parasite. Additionally, histogram equalization was applied to adjust the intensity distribution, improving contrast in regions with low variability (see Table 1).

Finally, a systematic exploration of all possible combinations of preprocessing techniques was conducted. Each color channel was tested independently and in combination with noise reduction and contrast enhancement techniques. This exhaustive analysis aimed to identify the preprocessing pipeline that optimally enhanced parasite features and visibility, minimized noise, and preserved structural details. The results of this stage provided a solid foundation for subsequent segmentation and classification steps.

### 2.3. Segmentation

Segmentation is a critical step in image analysis for identifying regions of interest with potential parasites. Since there is no standardized protocol for *Leishmania* spp., this research adopted methods proven in pathologies with similar visual diagnostic requirements, such as malaria and Chagas. Three effective segmentation techniques were selected from the scientific literature due to their computational efficiency and proven performance in microscopic image processing: Otsu’s method, local thresholding, and iterative global minimum segmentation (IGMS) [10,11,12,13,14].

Before applying segmentation, a padding process was performed on the images to avoid the loss of information in regions of interest near the edges, expanding the image margins with the average value of neighboring pixels. This step is essential to ensure that the cropped regions fully include the structures of interest. Otsu’s method automatically calculated a global threshold to distinguish between the background and parasites [15,16]. Local thresholding was implemented to adjust each image region’s threshold, a valuable technique for adapting to pixel value variability [17]. Finally, the IGMS method was employed to iteratively determine the optimal global threshold based on the average intensities of the region of interest and the background [18]. The experimental design of this stage involved evaluating a series of combinations of preprocessing techniques and segmentation methods. The objective was to identify the configuration that minimized the loss of relevant information in the final segmentation mask.

### 2.4. Feature Extraction

The feature extraction stage focused on identifying a representative set of features to analyze *Leishmania* spp. parasites in cutaneous leishmaniasis images. The selection of features was guided by their relevance in distinguishing parasites from background artifacts, as demonstrated in prior research on parasitic diseases [10,11,12,13,14,15,16,17,18,19,20]. Additionally, the process was aligned with the diagnostic protocol followed by professionals in conventional microscopy, which requires the clear visualization of three key structures—cytoplasm, nucleus, and kinetoplast—to confirm the presence of *Leishmania* spp. in direct smear samples. Based on this protocol, the extracted features were designed to characterize the parasite’s phenotypic traits in detail, ensuring consistency with clinical practices.

To comprehensively represent the phenotypic characteristics of the parasites, the features were grouped into three main categories: morphological, texture, and color. Morphological features, such as area, perimeter, compactness ratio, eccentricity, elongation, solidity, and Hu moments, were selected to describe the shape and physical structure of the parasites. These metrics are particularly useful for differentiating the distinct morphology of *Leishmania* amastigotes from background artifacts. For instance, compactness ratio and elongation help identify the parasite’s slender structure, while Hu moments provide robust shape descriptors that remain invariant to scale and rotation.

Texture features were extracted using the Gray Level Co-occurrence Matrix (GLCM) and included contrast, energy, dissimilarity, homogeneity, correlation, angular second moment (ASM), and entropy. These attributes capture intensity patterns within the segmented regions of the parasite, enabling the differentiation of granular and structural details. Features like contrast and entropy are particularly effective in highlighting the irregular intensity patterns present in the nucleus and kinetoplast, aiding in their precise identification.

Color features were derived from pixel values in the RGB color space, with statistics such as mean, standard deviation, skewness, and kurtosis. These features emphasize the color differences between the parasite and the background, which are particularly pronounced due to the characteristic staining patterns of Giemsa. The cytoplasm, nucleus, and kinetoplast exhibit distinct tinting compared to surrounding structures, making color features critical for their identification (see Table 2).

The extracted features provided a comprehensive representation of the parasite’s phenotypic traits, enabling precise classification. The consolidated dataset, containing feature vectors and corresponding labels for each crop, was saved in a CSV file. This dataset served as the foundation for creating three subsets: one with unbalanced classes and two balanced using different techniques. This alignment of feature selection with clinical diagnostic criteria enhances the reliability of the approach, bridging computational methods with conventional practices and ensuring the inclusion of diagnostically relevant structures. These decisions were further supported by prior findings on the importance of these attributes in biomedical image analysis [5,11,13].

### 2.5. Classification

The classification stage in our study was based on machine learning approaches applied to parasite detection in microscopic images [10,11,12,17,18,21]. Given the unique characteristics of our cutaneous leishmaniasis image dataset and the limited number of specific studies on cutaneous leishmaniasis, we opted for an experimental design using the Grid Search technique. This method is known for optimizing hyperparameters in machine learning models, enabling the systematic exploration and evaluation of different parameter combinations.

Grid Search was applied to three classic supervised machine learning algorithms. Support Vector Machine (SVM) was tested with different kernel configurations and by adjusting parameters such as “C” and “gamma”, following parameter settings reported in related studies [3,6,22]. Artificial Neural Network (ANN) was tuned by varying the network structure, including the number of layers, neurons, activation functions, dropout rates, and optimizers, based on configurations used in previous studies [23]. Random Forest (RF) was adjusted by manipulating variables such as the number of trees, maximum depth, minimum number of samples to split a node, and minimum number of samples for a leaf node, following parameters used in similar research [17] (see Table 3).

For the experimental design, the dataset obtained during the feature extraction stage was split into two sets: 70% for training and 30% for testing. This process was performed with three distinct data groups: one with unbalanced classes and two with balanced classes, using different techniques to ensure the model’s effectiveness in various scenarios. The experimental methodology aimed to evaluate various parameter combinations and determine the most efficient configurations for the classifiers.

### 2.6. Evaluation

The final phase of our study consists of the statistical evaluation of the developed system, aimed at verifying its functionality and determining its feasibility. In this stage, standard metrics in biomedical research were used to measure the system’s performance. These metrics include accuracy, sensitivity, specificity, precision, and F-Score, each providing a different perspective on how the system correctly classifies parasites and non-parasites in direct smear images. These metrics have been widely used in studies of diseases such as malaria, Chagas, and leishmaniasis, providing a solid reference framework for our evaluation [11,16,17,21].

Additionally, analytical tools such as confusion matrices and Receiver Operating Characteristic (ROC) curves were applied to the best classification model obtained for each classifier [17,18]. These tools are essential for providing a detailed and quantitative understanding of the model’s performance, particularly its ability to distinguish between parasites and non-parasites. It is important to note that the system’s evaluation was conducted at the crop level for the regions of interest within the direct smear examination images. The results of these metrics are crucial for determining the system’s effectiveness. They will lay the foundation for future research to expand the evaluation to a more comprehensive analysis, including full-field and patient-level evaluation. This advancement could represent a significant step in improving the conventional diagnostic process for cutaneous leishmaniasis.

## 3. Results

In this section, we present the key findings obtained throughout the project stages, based on developing a machine learning system for detecting the *Leishmania* spp. parasite in direct smear examination images. The significant results are described below, from image acquisition, preprocessing, segmentation, and feature extraction up to the classification and evaluation of the system’s performance, highlighting the effectiveness of the techniques employed.

### 3.1. Image Acquisition

**Figure 2:**
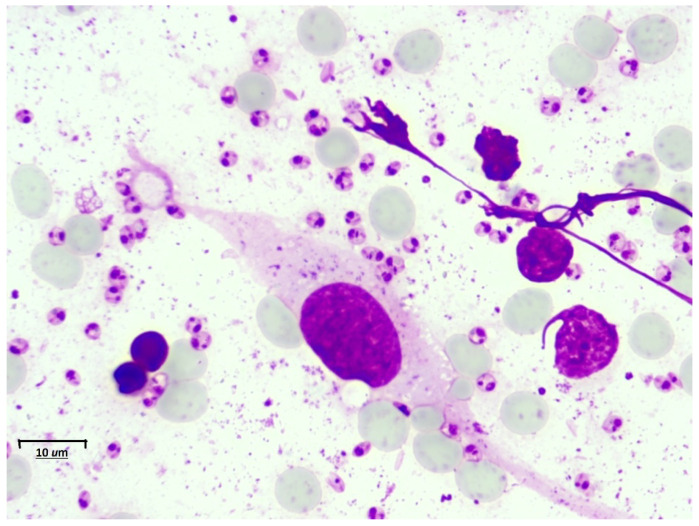
Original direct smear image of cutaneous leishmaniasis with the presence of Leishmania amastigotes. Scale bar 10 µm.

We successfully consolidated a database of cutaneous leishmaniasis images, consisting of 500 images in PNG format with a resolution of 2592 × 1944 pixels. The dataset was organized into 50 folders, each representing direct smear examination samples from different patients to ensure sufficient variability in the collected data. The selection of samples and the digitization of the slides were carried out under a protocol developed in collaboration with specialists from the Clinical Laboratory of the E.S.E Hospital Universitario Centro Dermatológico Federico Lleras Acosta. This protocol allowed for standardized image capture, ensuring that each sample was digitized under optimal lighting and focus conditions (see Figure 2).

Parasite labeling in the images was performed using the LabelImg software tool, where we successfully identified and labeled 7905 *Leishmania* spp. parasites. This process was carried out in collaboration with experts on cutaneous leishmaniasis diagnosis, ensuring the accuracy of the data for subsequent use in the research. The labeling details for each parasite were captured using bounding boxes, which allowed for the extraction of height and width values and the x and y coordinates of the parasite’s exact location within the image. This information was recorded in JSON files corresponding to each image. The meticulous acquisition and labeling of these images provided a solid foundation for the subsequent phases of the project, ensuring the integrity and quality of the processed information (see Table 4).

### 3.2. Preprocessing

We conducted an experimental design in the preprocessing phase to evaluate different noise reduction and contrast enhancement technique combinations. This process focused on optimizing the visualization and analysis of images to detect *Leishmania* spp. parasites accurately. We began preprocessing by decomposing the original images into their respective R, G, B, Grayscale, H, S, and V color channels. This separation into individual channels was crucial for highlighting distinct aspects of the parasites, depending on the specific information provided by each channel, allowing for more detailed and focused visualization of parasitic features.

Subsequently, three noise reduction techniques were implemented: a median filter with a 3 × 3-pixel kernel, which effectively removed minor noise points while preserving the sharpness of the parasites’ structural edges. The second filter was a Gaussian filter with a 7 × 7-pixel kernel and a standard deviation of 3, which smoothed the images, reducing random noise and improving overall clarity. The third filter applied morphological closing operations using a 7 × 7-pixel rectangular structuring element, which closed gaps in visible structures while preserving the integral shape of the parasites.

**Figure 3:**
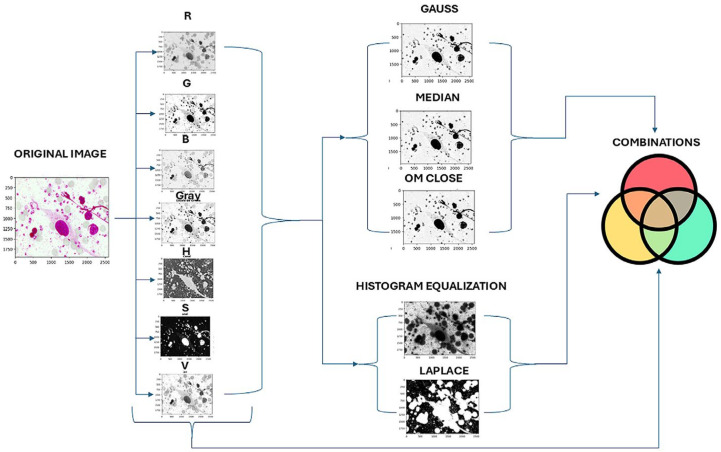
Experimental design of preprocessing.

During initial tests, we also identified that the contrast enhancement techniques of Laplacian filtering and histogram equalization were not beneficial for our images. These techniques created distortions and opaque areas that could obscure parasites in the final mask, so they were excluded from the experimental design. Twenty-eight possible combinations were tested, evaluating each color channel alone and in combination with noise reduction techniques. This exploration was crucial for identifying the most efficient configuration for preprocessing cutaneous leishmaniasis images. The best preprocessing combination was selected in the segmentation stage (see Figure 3).

### 3.3. Segmentation

**Figure 4:**
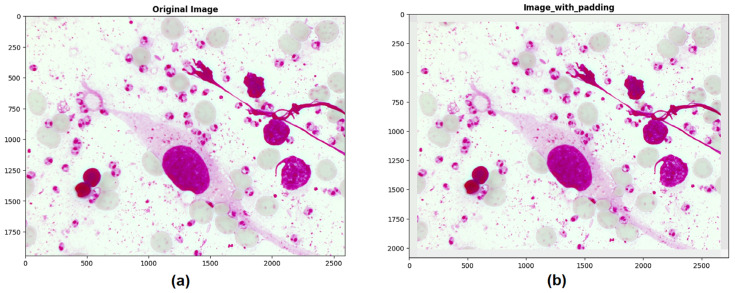
(**a**) Original image, (**b**) Image with 70-pixel border padding.

For this stage, a padding technique was necessary to extend the border of each image by 70 pixels using the average value of neighboring pixels. This step was performed to avoid errors while cropping regions of interest near the edges and to ensure that the crops and bounding boxes were not cut off from the original image (see Figure 4).

Subsequently, the pixel distribution in each color channel (R, G, B, Grayscale, H, S, V) was visually analyzed through the average histograms of the 500 images. This step provided direct information about the images’ contrast, exposure, and average color, which was crucial for selecting preprocessing and segmentation techniques.

**Figure 5:**
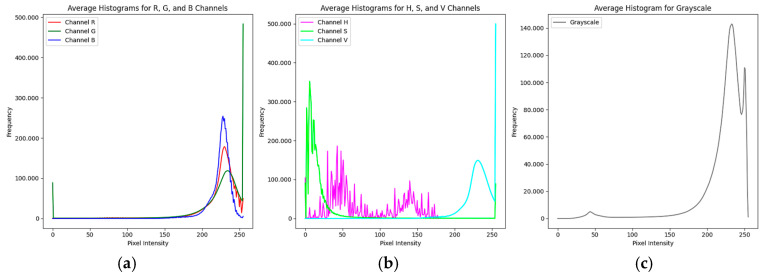
Average histograms of 500 images in color channels: (**a**) Color space (R, G, B), (**b**) Color space (H, S, V), (**c**) Grayscale.

The average histograms in the R, G, B, and Grayscale channels of the direct smear microscopy images stained with Giemsa reveal notable pixel intensity distribution characteristics. They show predominant peaks at higher intensities, around intensity 230, suggesting a significant presence of background regions with light tones. The absence of peaks in the low-intensity areas indicates that darker regions, potentially representing the nuclei and kinetoplasts of the parasites, are less frequent compared to the lighter background. This result reflects the expectation that the areas of interest (parasites) are darker and less prevalent than the lighter background in these microscopy images due to the specific characteristics of these images (see Figure 5a,c).

The average histograms of the H, S, and V channels provided insights into the color and brightness distribution in the images. The H channel, which indicates color hue, shows a distribution with smaller peaks, suggesting a variety of tones present in the samples. This finding could reflect different elements within the images, such as parasite structures and some noisy objects. The S channel, which measures color saturation, presents low to medium saturation peaks, likely corresponding to the regions of interest as potential parasites. Finally, the V channel, which shows intensity or brightness, has high peaks at the far right, indicating very bright areas, possibly related to artifacts in the image background (see Figure 5b).

Once the padding process and histogram visualization were completed, three segmentation methods were selected and implemented to identify potential *Leishmania* spp. parasite candidates. Initially, Otsu’s method was applied, automatically calculating an optimal global threshold to differentiate between background pixels and regions of interest. This method proved particularly effective when there was a high contrast between the background and the parasite. The second method was local thresholding, where a 71 × 71-pixel window and a “mean” approach were used to calculate the threshold for each local region of the image, allowing it to adapt to pixel value variability across regions. The third method implemented was Iterative Global Minimum Screening (IGMS), configured with an initial threshold of 127, which was iteratively modified based on the average intensities of the region of interest and the background to find the optimal global threshold.

**Figure 6:**
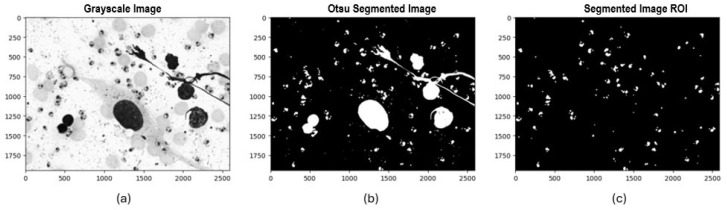
Experimental design process applying Otsu segmentation: (**a**) Grayscale image, (**b**) Binarized image with Otsu, (**c**) Final ROI mask.

**Figure 7:**
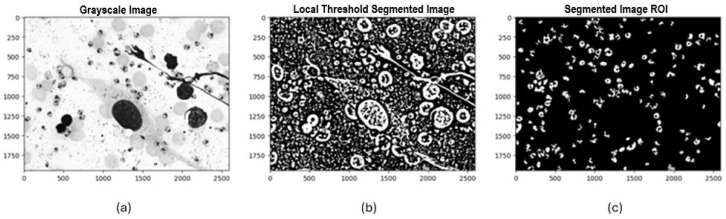
Experimental design process applying local threshold segmentation: (**a**) Grayscale image, (**b**) Binarized image with Local Threshold, (**c**) Final ROI mask.

**Figure 8:**
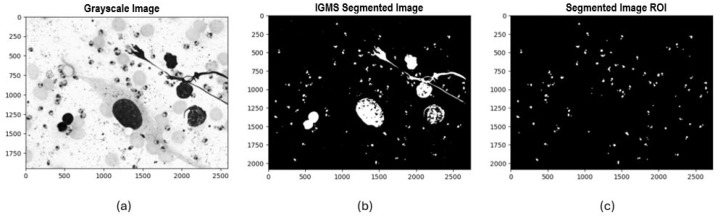
Experimental design process applying IGMS segmentation: (**a**) Grayscale image, (**b**) Binarized image with IGMS, (**c**) Final ROI mask.

Following this process, we evaluated 84 resulting combinations of preprocessing techniques (7 color channels alone and in combination with each noise reduction filter) for each of the three segmentation methods with binarization techniques (Otsu, local thresholding, IGMS) to determine the most efficient configuration that preserved the most information in the final mask. Each combination was evaluated by generating a binary image that differentiated the background and regions of interest. Afterward, an initial mask cleanup was performed to remove large objects greater than 3000 pixels and small ones smaller than 100 pixels that did not correspond to potential parasites. Following this process, a count and identification of the number of parasites present in the final mask was carried out (see Figure 6, Figure 7 and Figure 8).

Example of the implementation of experimental design combinations applying segmentation with Otsu:

Example of the implementation of experimental design combinations applying local thresholding segmentation:

Implementation of experimental design combinations applying IGMS segmentation:

Subsequently, to differentiate between actual parasites and artifacts, we extracted the central point of each object in the mask and compared it with the location of parasites labeled by experts. Objects whose centroids were within a proximity range of 50 pixels from the labeled point were classified as parasites. Finally, 140 × 140-pixel crops were taken from the original image, considering the centroids of the objects in the final mask to consolidate two subgroups of images: one containing parasites and the other containing artifacts (non-parasites), based on expert labeling.

Finally, the best results from the combinations of preprocessing and segmentation in the experimental design were consolidated, yielding significant outcomes. The combination of IGMS with the R channel and the morphological closing operator proved to be the most effective, detecting 95.98% of parasites and generating only 15,582 artifacts. Otsu’s method with the S channel and a Gaussian filter detected 92.35% of parasites with 22,184 artifacts. In contrast, the local threshold with the S channel and a Gaussian filter had the lowest performance, detecting 85.11% of parasites and generating 87,733 artifacts. These results highlight the superiority of the IGMS + R CHANNEL + CLOSE combination in terms of accuracy and efficiency in parasite segmentation in the final mask (see Table 5).

### 3.4. Feature Extraction

The feature extraction stage focused on the crops obtained during the segmentation process. Based on the protocol and expert criteria for confirming the presence of the parasite in the sample, it was necessary to identify the three phenotypic structures of the parasite: the nucleus, kinetoplast, and cytoplasm. Accuracy in this phase was crucial to confirm the presence of the parasites. When analyzing the crops, three predominant shades were identified that facilitated the discrimination of the parasite structures: a light tone corresponding to the background of the crop, where no parasite structures are present; a medium tone representing the parasite membrane and cytoplasm; and a dark tone indicative of the nucleus and kinetoplast, which are essential parts of the parasite.

**Figure 9:**
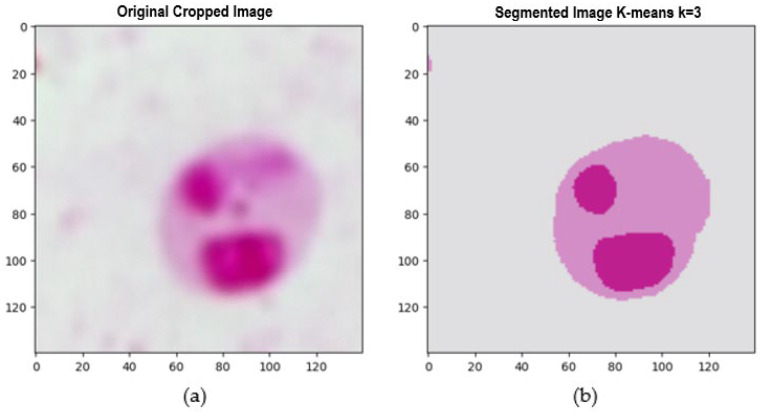
(**a**) Original cropped image, (**b**) K-means segmented image.

**Figure 10:**
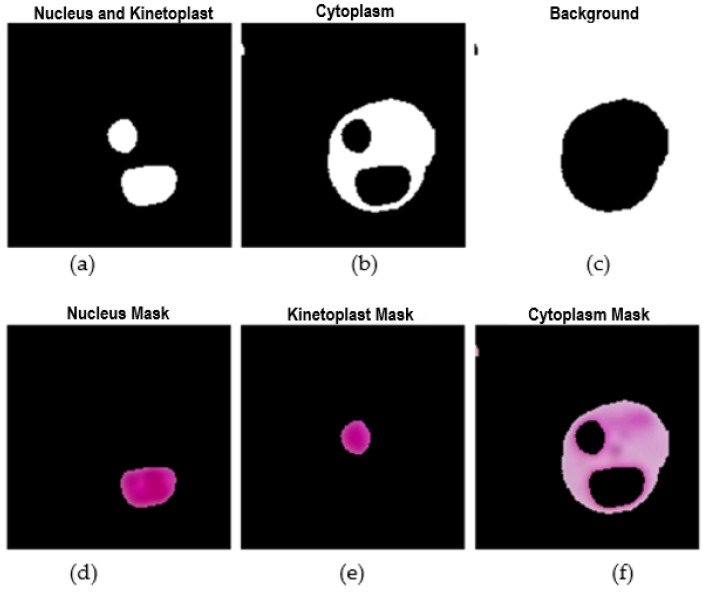
Structure extraction process using K-means segmentation: (**a**) Nucleus and kinetoplast mask, (**b**) Cytoplasm mask, (**c**) Background mask, (**d**) Final nucleus mask, (**e**) Final kinetoplast mask, (**f**) Final cytoplasm mask.

To effectively separate these structures, a segmentation technique at the crop level was implemented using the k-means method. This approach allowed for segmenting the images based on the three tones, which was crucial for the precise extraction of each structure individually. This differentiation between the nucleus, kinetoplast, and cytoplasm facilitated the detailed extraction of features from each structure. Figure 9 and Figure 10 illustrate the segmentation and feature extraction process through visual examples showing each phenotypic structure of the *Leishmania* spp. and how the parasite was isolated, enabling a clear and precise representation of the parasite features in the analyzed crops.

The process of extracting and individualizing the three phenotypic structures of the *Leishmania* spp. a parasite is visualized below:

In terms of feature extraction, a total of 96 features were applied to each crop, distributed into three main groups: 13 morphological features (area, perimeter, compactness ratio, eccentricity, elongation, solidity, and 7 Hu moments), 12 color features (mean value, standard deviation, skewness, kurtosis) for the R, G, and B channels, and seven texture features (contrast, energy, dissimilarity, homogeneity, correlation, angular second moment (ASM), and entropy) for each of the three parasite structures. The results of this stage were consolidated into a CSV file containing the feature vectors and corresponding labels for each crop.

This CSV file generated three datasets: one with unbalanced classes (parasites and non-parasites), which included all the artifacts segmented in the final segmentation mask, and two balanced datasets. Two different approaches were used to achieve class balance in the balanced datasets. In one dataset, dimensionality was reduced by randomly selecting samples from the non-parasite class to match the number of samples in the parasite class. In the other dataset, the k-nearest neighbors (k-NN) algorithm was used, which was trained with the parasite class feature set to select the same number of non-parasites, considering the artifacts with the most similar features to the parasite class (see Table 6).

### 3.5. Classification and Evaluation

The experimental design for the classification stage was developed based on the grid search technique. This methodology is widely recognized in machine learning and is used to optimize model hyperparameters through an exhaustive search and evaluation of all possible combinations. This classification process was applied to three classic supervised learning algorithms: Support Vector Machine (SVM), Artificial Neural Network (ANN), and Random Forest (RF). Each algorithm was subjected to an exploration of various hyperparameter combinations to determine the best configuration for each.

Notably, the grid search technique included an internal 5-fold cross-validation, significantly improving the models’ robustness. For this cross-validation, the dataset obtained in the feature extraction stage was split into two sets: 70% for training and 30% for testing. This process was performed with three different datasets: one with unbalanced classes, one with balanced data by random selection, and the third using the K-Nearest Neighbors (KNN) technique. The experimental approach explored 604 parameter combinations to identify the most effective classifier. It is also worth mentioning that all three datasets were evaluated using the same test set, ensuring a fair and accurate comparison of the models across the three datasets.

Below, we present a detailed summary of the best hyperparameter combinations for each classifier and the model’s performance on the different datasets (Unbalanced, Randomly Balanced, and KNN Balanced). These tables show the obtained metrics, including accuracy, sensitivity, specificity, precision, and F-score, allowing for a direct comparison of the performance of the three machine learning models: Support Vector Machine (SVM), Artificial Neural Network (ANN), and Random Forest (RF) (see Table 7 and Table 8).

Results of the experimental design by model, total number of combinations, and best combination:

Results of the best parameter combination in the Unbalanced dataset (7587 Parasites and 15,576 Random Non-parasites): In the unbalanced dataset, the SVM model showed high sensitivity (95.00%) but low specificity (74.68%), indicating a high number of false positives. The ANN model displayed a more balanced performance with a sensitivity of 92.36% and specificity of 81.12%, with an AUC of 0.87, demonstrating robust discriminatory capability. On the other hand, the RF model had the lowest sensitivity (79.28%) but the highest specificity (85.21%), suggesting better handling of false positives, although its AUC was the lowest (0.82).

Results of the best parameter combination in the Randomly Balanced dataset (7587 Parasites and 7587 Random Non-parasites): All models improved their performance in the randomly balanced dataset. SVM increased its accuracy (83.96%) and AUC (0.87). ANN showed the best accuracy (87.26%) and the highest F-Score (82.08%), with an AUC of 0.88, indicating balanced performance. RF also improved significantly, with a specificity of 88.63% and an accuracy of 76.55%, although its AUC was 0.83, reflecting an improvement in class discrimination compared to the unbalanced dataset.

Results of the best parameter combination in the KNN Balanced dataset (7587 Parasites and 7587 Nearest Non-parasites): In the KNN Balanced dataset, SVM stood out with the best accuracy (90.07%), sensitivity (91.87%), specificity (89.21%), and an AUC of 0.91, indicating excellent discriminatory capability for both classes. ANN also showed robust performance, with specificity of 90.14%, accuracy of 84.12%, and an AUC of 0.89. RF achieved a good balance between sensitivity (89.64%) and specificity (89.91%), with an AUC of 0.90, emphasizing the effectiveness of balancing the data with KNN in improving the accuracy of the three models.

**Figure 11:**
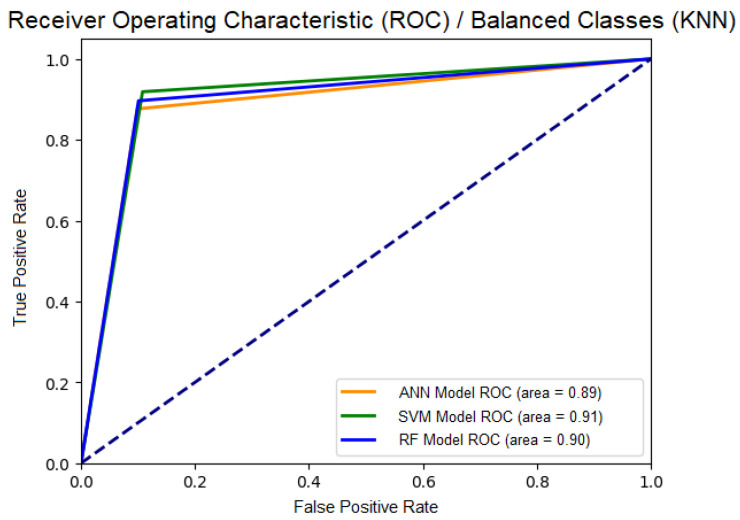
ROC of SVM, ANN, RF Models with “KNN Balanced” data.

Additionally, we graphically consolidated the results using the Receiver Operating Characteristic (ROC) curves for the three models (SVM, ANN, RF) with “KNN Balanced” data in the best results (see Figure 11).

In this graph, it can be observed that all three models exhibit similar performance. The SVM model shows the highest area under the curve (AUC) at 0.91, followed by the RF model with an AUC of 0.90 and the ANN model with an AUC of 0.89. A higher AUC value indicates better discrimination ability of the model between positive and negative classes—in our case, parasites and non-parasites. The fact that the three curves are very close and have high AUC values suggests that all three models can accurately classify cases in this KNN-balanced dataset. This fact may indicate that the balancing technique used positively impacted the models’ predictive capability.

## 4. Discussion

In this project, we presented an approach that utilizes machine learning algorithms to detect *Leishmania* spp. parasites in microscopy images. During this process, we consolidated a database with 500 direct smear images containing 7905 parasites. This database allowed us to train machine learning models with realistic variability, ensuring solid results in subsequent stages. The importance of this image database lies in its ability to accurately reflect the diversity of clinical samples encountered in field medical practice.

Moreover, the preprocessing stage played a crucial role in analyzing image quality, with two significant findings. The first was identifying that applying contrast enhancement techniques distorted and generated opaque areas in the image, leading to the loss of parasites when applying the segmentation method. For this reason, these techniques were excluded from the original experimental design combinations, focusing instead on color channel combinations with noise reduction techniques. The second important finding is related to the relevance of the R channel (red channel), both alone and in combination with the morphological closing filter, in discriminating parasitic structures. This channel proved essential in highlighting the phenotypic characteristics of the parasite when combined with the segmentation methods.

In this study, three conventional segmentation methods were employed: Otsu, local thresholding, and Iterative Global Minimum Screening (IGMS). These techniques were selected for their simplicity, computational efficiency, and widespread use in research related to parasitic diseases such as malaria and Chagas [11,12,14]. Although deep learning-based techniques, such as U-Net, offer significant advantages in segmentation, their implementation was limited by the dataset size and available computational resources. The conventional methods proved effective within the scope of this study. For instance, IGMS detected 95.98% of parasites in the final mask. However, these methods are sensitive to variability in acquisition conditions, such as lighting, resolution, and staining, which can impact their performance. These limitations were mitigated through specific preprocessing techniques, such as noise reduction and contrast enhancement, which optimized image quality before segmentation.

On the other hand, deep learning techniques like U-Net stand out for their ability to learn complex patterns and generalize better in highly variable scenarios [21]. However, their use requires large volumes of annotated data and advanced computational resources, which exceeded the capabilities of this study. Nonetheless, the conventional techniques provided a practical and efficient solution, especially with limited technological resources. The choice of Otsu, local thresholding, and IGMS was motivated by their ease of implementation in diagnostic systems that do not rely on specialized hardware. These techniques are viable for regions where cutaneous leishmaniasis is endemic, providing an accessible and low-cost tool. We plan to expand the dataset to include greater sample diversity and explore advanced deep learning models such as U-Net for future work. This will allow us to evaluate their capacity to improve segmentation and deliver more robust results in microscopic images of cutaneous leishmaniasis.

The feature extraction stage was crucial for phenotypically describing the parasite. Initially, it was established that three main structures must be visible to detect a parasite: the nucleus, kinetoplast, and cytoplasm. Therefore, alternative methods, such as k-means, were used to segment and extract these structures, ensuring proper characterization individually. This method performed excellently, consolidating morphological, texture, and color features so that the machine learning models could accurately identify the differences between parasites and non-parasites. Once the phenotypic dataset for both classes was consolidated, three datasets were created: Unbalanced, Randomly Balanced, and Balanced with K-Nearest Neighbors (KNN). The implementation of KNN was particularly relevant, as it showed the best results in the experimental design of the classifiers. Balancing the data was crucial to avoid bias in training and ensure accurate classification of the parasites.

In the classification stage, it is essential to note that to optimize classifier performance, we applied a technique reported in previous studies known as “grid search”. This technique played a crucial role by allowing each model to optimize its parameters, ensuring that the models reached their maximum capacity to identify parasites with excellent levels of sensitivity and specificity. This optimization technique contributed to the models’ effectiveness. It ensured that cross-validation techniques were applied during training, resulting in more robust and effective models for parasite detection in direct smear images.

The results obtained in our classification and evaluation stage corroborate previous research findings, confirming the viability of machine learning in diagnosing parasitic diseases. Comparing our metrics with those reported in similar studies reinforces our findings’ relevance and highlights our methodology’s consistency. For example, the study by Zare et al. (2022) [7], which implemented the Viola–Jones algorithm with a feature extraction methodology, created integral images for segmentation and used AdaBoost for feature selection and classifier training, achieved a sensitivity of 71% and a specificity of 52% in detecting amastigotes in direct smear images of cutaneous leishmaniasis. Similarly, Isaza-Jaimes et al. (2020) [24] applied a methodology starting with image preprocessing, followed by a region of interest selection and gradient module implementation using polar maps for classification, achieving an accuracy of 78.79% in detecting *Leishmania* spp. parasites in visceral leishmaniasis direct smear images.

Other deep learning approaches, such as that of Górriz M. (2018), implemented an automated system based on a U-net convolutional neural network to segment *Leishmania* parasites and classify them into promastigotes, amastigotes and attached parasites, achieving an accuracy of 75.7% and a sensitivity of 82.3% in detecting amastigotes of cutaneous leishmaniasis acceptable results but with room for improvement given the small dataset of 45 images used for the method [21]. With the SVM as the best classifier, our method achieved a sensitivity of 91.87% and a specificity of 89.21% in detecting *Leishmania* spp. parasites at the crop level in direct smear images. These results set a precedent and provide a starting point for improving the techniques and methods implemented.

On the other hand, Sadeghi et al. (2024) introduced a deep learning-based model for detecting amastigotes using pre-trained architectures such as ResNet and MobileNet. Their approach achieved an average accuracy of 95.7% and a sensitivity of 97.2%, highlighting the potential of fine-tuning and deep learning in telemedicine applications. However, this approach relies on large, well-balanced datasets, which can pose a challenge in resource-limited settings [25]. Our study complements these findings by demonstrating that explainable methods with less dependency on large volumes of data can achieve competitive performance, especially in environments where model interpretability and traceability are crucial.

Finally, Tekle et al. (2024) presented DeepLeish, a system that uses YOLOv5, Faster R-CNN, and SSD to detect parasites in microscopic images. YOLOv5 demonstrated the best performance with a mAP of 73%, precision of 68%, and a recall of 69%, highlighting its potential for real-time detection despite its moderate accuracy [26]. However, the results could benefit from significant improvements in preprocessing and segmentation to enhance the detection of parasites. In contrast, our approach, based on IGMS combined with the R channel and morphological closing, achieved a 95.98% recovery rate of parasites in the generated masks, with a sensitivity of 91.87% and a specificity of 89.21%. This demonstrates that a carefully designed preprocessing and segmentation pipeline can achieve a level of performance that rivals or even surpasses modern detection architectures, particularly when working with high-resolution images and limited datasets.

Overall, our work serves as a bridge between explainable classical methods and advanced deep learning models, ensuring robust performance while offering an accessible and adaptable solution for clinical scenarios with technological or data limitations. Future research could explore the integration of modern architectures such as YOLOv8 or U-Net, applying transfer learning to build upon the strong foundations of this study. Additionally, expanding the dataset and conducting clinical validation will be crucial to further optimize the system for real-world applications.

Although the results obtained are promising, this study has some limitations. First, the model validation was performed at the cropped image level, which does not fully reflect clinical diagnostic conditions in full samples or at the patient level. Additionally, the robustness of the developed machine learning models was primarily validated using a standardized dataset acquired under controlled conditions. This dataset consists of images captured with a single type of microscope (Olympus CX43) and prepared using uniform staining protocols, ensuring consistency in resolution, staining, and lighting conditions. While this uniformity provided a solid foundation for the study, it limits the models’ generalizability to data from other microscopes or preparation techniques.

To address these limitations, future work should expand the dataset to include images obtained from diverse microscope models and slides prepared with varying staining protocols. These additions would enable the models to learn from a broader range of variations, enhancing their adaptability to real-world clinical settings. Furthermore, methodologies to improve the models’ ability to generalize across datasets with differing characteristics should be explored. Despite the current dataset’s limitations, preprocessing steps such as noise reduction and contrast enhancement were applied to mitigate variability within the collected data. These steps aimed to normalize the images and minimize the impact of minor variations, thereby improving the model’s robustness within the scope of the study. However, comprehensive testing on external datasets is necessary to fully evaluate the models’ performance and reliability in diverse clinical scenarios.

Another limitation is the lack of extensive clinical validation in real-world settings, which is essential to evaluate the system’s performance in field conditions. Finally, while the models demonstrated high sensitivity and specificity, their implementation on mobile devices or low-cost equipment still requires technical adjustments and further evaluation. These limitations highlight the need for future studies to expand the database, assess the system’s performance in clinical environments, and optimize the technology for practical use in resource-limited areas.

## 5. Conclusions

This study developed a machine learning-based approach for detecting *Leishmania* spp. parasites in direct smear microscopy images, providing significant support for the diagnosis of cutaneous leishmaniasis. By consolidating a robust database of 500 images containing 7905 annotated parasites, we trained and optimized machine learning models that demonstrated high effectiveness in both segmentation and classification tasks.

Our experimental results demonstrate that preprocessing techniques leveraging the red (R) channel, combined with noise reduction methods and segmentation via Iterative Global Minimum Screening (IGMS), successfully recovered approximately 95% of parasites in the final segmentation mask. The precise extraction of key parasite structures nucleus, kinetoplast, and cytoplasm facilitated detailed phenotypic characterization, which significantly enhanced classification performance.

Among the classifiers tested, the Support Vector Machine (SVM) achieved the best results, with an average performance of 90% across precision, sensitivity, specificity, and accuracy metrics. These findings confirm that machine learning algorithms can significantly improve the detection and classification of parasites in microscopy images, offering a promising and accessible technological solution for diagnosing cutaneous leishmaniasis, particularly in resource-constrained settings.

However, this study has some limitations. The analysis was conducted at the crop level, which does not fully reflect the complexity of clinical diagnostic workflows that involve whole-slide evaluations. Future work should aim to validate the system in real clinical environments, incorporating full-field microscopy images and patient-level data to ensure broader clinical applicability. Additionally, expanding the dataset to include images from different microscopes and staining protocols will be crucial to improving the system’s generalizability.

Furthermore, integrating advanced deep learning architectures, such as U-Net or YOLOv8, and exploring their potential to automate image analysis could enhance the system’s precision and scalability. The development of mobile-based diagnostic tools also holds promise for increasing accessibility in remote or resource-limited areas.

In conclusion, this study makes a significant contribution to the field of diagnostic medicine by establishing a robust foundation for applying machine learning to the detection of cutaneous leishmaniasis. The proposed approach demonstrates the potential to improve diagnostic accuracy and efficiency, ultimately supporting better patient outcomes and advancing the integration of technology in medical practice.

## Figures and Tables

**Table 1:** Benefits and limitations of preprocessing techniques.

Benefits and Limitations of Preprocessing Techniques			
Group of Techniques	Technique	Benefits	Limitations
**Noise** **Reduction**	**Median** **Filtering**	Removes salt-and-pepper noise effectively. Preserves edges and morphological integrity of parasites.	Limited effectiveness for complex noise patterns.
	**Gaussian** **Filtering**	Reduces random noise, enhancing the clarity of the image. Smooth high-frequency noise while maintaining general structure.	Slightly blurs fine details, such as the kinetoplast.
	**Morphological Closing**	Bridges small gaps in parasite contours, improving continuity.	Overuse may merge structures, potentially distorting parasite shapes.
**Contrast** **Enhancement**	**Laplacian** **Filtering**	Highlights edges, improving the visibility of structural details.	Introduces artifacts and over-enhancement in certain regions, leading to loss of critical information.
	**Histogram Equalization**	Improves contrast in low-contrast regions, making darker regions more distinguishable.	Can obscure parasite features by introducing areas of excessive brightness or darkness.

**Table 2:** Feature sets.

Feature Sets	
Category	Features
Color	Mean value
	Standard deviation
	Skewness
	Kurtosis
Texture	Contrast
	Energy
	Dissimilarity
	Homogeneity
	Correlation
	Angular second moment (ASM)
	Entropy
Morphological	Area
	Perimeter
	Compactness ratio
	Eccentricity
	Elongation
	Solidity
	Hu moments

**Table 3:** Model parameters.

Model	Parameters	Parameter Values
SVM	C	(0.1, 1, 10, 100)
	Gamma	(0.001, 0.01, 0.1, 1)
	Kernel	(linear, polynomial, radial, sigmoid)
ANN	Activation Function	(“relu”, “sigmoid”, “tanh”)
	Dropout	(0.0, 0.2, 0.5)
	Number of Layers	(1, 2, 3, 4)
	Number of Neurons	(32, 64, 128, 256)
	Optimizer	(“adam”, “sgd”, “rmsprop”)
RF	Number of Trees	(100, 200, 300)
	Maximum Depth	(None, 10, 20, 30)
	Min Samples Split	(2, 5, 10)
	Min Samples Leaf	(1, 2, 4)

**Table 4:** Database description.

Database	
Description	Results
Total images	500
Image dimensions	[1944 × 2592]
Image format	“PNG”
Labeling format	“JSON”
Total labeled parasites	7905

**Table 5:** Best results from experimental design of preprocessing—segmentation.

Experimental Design Preprocessing and Segmentation—Best Results			
**Combination**	OTSU + CHANEL S + GAUSS	LOCAL THRESHOLD + CHANEL S + GAUSS	IGMS + CHANEL R + CLOSE
Parasite Count	7300	6728	7587
Parasite Percentage	92.35%	85.11%	95.98%
Non-Parasite Objects (Artifacts)	22,184	88,733	15,582
Final Mask Objects	29,473	95,417	23,169
Total Segmented Objects	48,578	1,440,879	35,291

**Table 6:** Consolidation of datasets.

	Training Set (70%)			Test Set (30%)		
Dataset	Parasites	Non-Parasites	Size	Parasites	Non-Parasites	Size
Unbalanced	5347	10,867	16,214 Objects 96 Features	2240	4709	6949 Objects 96 Features
Randomly Balanced	5347	5347	10,694 Objects 96 Features	2240	4709	6949 Objects 96 Features
KNN Balanced	5347	5347	10,694 Objects 96 Features	2240	4709	6949 Objects 96 Features

**Table 7:** Results of experimental design parameter combinations in the classifiers.

Model	Best Parameter Combination with Grid Search
SVM	C: 10
	Gamma: 0.01
	Kernel: rbf
ANN	Activation Function: relu
	Dropout: ‘0.5’
	Number of Layers: 2
	Number of Neurons: 256
	Optimizer: Adam
RF	Number of Trees: 300
	Maximum Depth: None
	Minimum Samples per Node: 5
	Minimum Samples per Leaf: 1

**Table 8:** Results of SVM, ANN, RF Models in Unbalanced, Randomly Balanced, and KNN Balanced Datasets.

Dataset	Model	Accuracy (%)	Recall (%)	Specificity (%)	Precision (%)	F-Score (%)	AUC
Unbalanced	SVM	81.23	95.00	74.68	64.09	76.54	0.85
	ANN	84.74	92.36	81.12	69.94	79.60	0.87
	RF	83.30	79.28	85.21	71.84	75.38	0.82
Randomly Balanced	SVM	83.96	93.70	79.33	68.32	79.02	0.87
	ANN	87.26	90.53	85.70	75.08	82.08	0.88
	RF	85.20	77.99	88.63	76.55	77.26	0.83
KNN Balanced	SVM	90.07	91.87	89.21	80.20	85.64	0.91
	ANN	89.33	87.63	90.14	80.88	84.12	0.89
	RF	89.82	89.64	89.91	80.86	85.03	0.90

## Data Availability

Research data will be openly available in zenodo (https://zenodo.org (accessed on 16 December 2024)).
